# The E5 protein of the human papillomavirus type 16 down-regulates HLA-I surface expression in calnexin-expressing but not in calnexin-deficient cells

**DOI:** 10.1186/1743-422X-4-116

**Published:** 2007-10-30

**Authors:** Myriam Gruener, Ignacio G Bravo, Frank Momburg, Angel Alonso, Pascal Tomakidi

**Affiliations:** 1 Division of Cell Differentiation, German Cancer Research Center, Heidelberg, Germany; 2 Division of Genome Modifications and Carcinogenesis, German Cancer Research Center, Heidelberg, Germany; 3 Division of Molecular Immunology, German Cancer Research Center, Heidelberg, Germany; 4 Department of Dental Medicine, University of Heidelberg, Heidelberg, Germany; Germany; 5 Experimental Molecular Evolution. Institute for Evolution and Biodiversity, University of Muenster, Muenster, Germany

## Abstract

The human papillomavirus type 16 E5 protein (HPV16 E5) down-regulates surface expression of HLA-I molecules. The molecular mechanisms underlying this effect are so far unknown. Here we show that HPV16 E5 down-regulates HLA-I surface expression in calnexin-containing but not in calnexin-deficient cells. Immunoprecipitation experiments reveal that calnexin and HPV16E5 can be co-precipitated and that this association depends on the presence of a wild-type first hydrophobic region of E5. When an E5 mutant (M1) in which the first putative transmembrane helix had been disrupted was used for the transfections calnexin-E5 co-precipitation was strongly impaired. In addition, we show that the M1 mutant is only able to marginally down-regulate HLA-I surface expression compared to the wild-type protein. Besides, we demonstrate that E5 forms a ternary complex with calnexin and the heavy chain of HLA-I, which is mediated by the first hydrophobic region of the E5 protein. On the basis of our results we conclude that formation of this complex is responsible for retention of HLA-I molecules in the ER of the cells.

## Introduction

Epidemiological analyses have demonstrated a close association between infection of certain human papillomavirus (HPV) species within the *Alphapapillomavirus *genus and malignant growth of the human cervix epithelium [1-3], as HPV sequences have been found in virtually all cervical cancers [4]. HPV types associated to cervical cancer are phenomenologically named as "high-risk HPVes", and about 70 % of the HPV sequences isolated from cervical lesions have been identified as being HPV type 16 or 18 [5,6]. High-risk HPV infection of the stratified epithelium occurs first in the basal cell layer, where transcription of the early genes E5, E6 and E7 takes place [7,8]. Upon upwards migration towards more superficial layers and concomitant differentiation of the infected keratinocyte, the late genes of the virus are expressed leading to the formation of viral particles and their release upon cell death.

During evolution the arms race between papillomaviruses (PVes) and their hosts has resulted in parallel selection of cellular mechanisms aiming to clear viral infection, such as inhibition of cellular apoptosis or uncoupling of the normal proliferation/differentiation program of the epithelium on the one hand, and in selection of viral mechanisms aiming to hamper cellular reaction directed to clear infection on the other. In this context, several molecular interactions between the oncogenes HPV16 E5, E6 and E7 and different apoptotic pathways have already been identified [9]. E6 and E7 modulate apoptosis by binding and inactivating p53 and the product of tumour suppressor gene Rb1 respectively [10,11], thereby deregulating the cell cycle. E5 impairs ligand-mediated apoptosis by reducing the amount of surface CD95 proteins or inhibiting the formation of the DISC complex [12], and affects the normal functioning of a number of membrane associated proteins, probably by modifying the composition and the interactions in the cell membranes [13]. Another mechanism evolved in certain PVes proceeds through down-modulation of the host adaptive immunoresponse. In this context it should be mentioned that whereas antibodies against E6 and against E7 have been found in blood of infected patients [14,15], no antibodies against E5 have been so far detected [16-18].

Using cellular systems it has been shown that HPV16 E5 expression results in down-regulation of cell surface expression of HLA-I and HLA-II molecules [19-22]. This down-regulation might result in diminished antigen-presentation and decreased adaptive immunoresponse of the host. Interestingly, a reduced expression of HLA-I molecules has also been detected in squamous cell carcinomas of the cervix compared to uninfected epithelium [23]. The decrease in HLA-I surface expression seems to be mediated by a failure in the HLA-complex transport systems to the cell membrane, which accumulate instead in the endoplasmic reticulum [22,24]. The molecular mechanisms that lead to this impaired intracellular trafficking are unknown. Recently it has been shown that HPV16 E5 may co-precipitate with the heavy chain of HLA-I in cells over-expressing the E5 protein [21]. Nevertheless, no biological evidence has been presented demonstrating that this association is responsible for the down-regulation of HLA-I surface expression. Thus, the intimate mechanisms responsible for the reduced amount of HLA-I molecules at the cell surface remain still elusive.

Calnexin is a chaperone that plays a major role in HLA-I maturation and surface transport [25-27]. Based on the observation that in cervical cancer lesions the expression of calnexin is deregulated [28], we hypothesyse that this chaperone is involved in the E5-mediated down-regulation of HLA-I surface expression. In this communication we present experimental evidence showing that HPV16 E5 down-regulates cell surface expression of HLA-I in calnexin-expressing but not in calnexin-deficient cells. We further show that E5 associates and co-localizes with calnexin and forms a ternary complex with the heavy chain of HLA-I molecules. Further, we show that E5 mutants unable to bind calnexin fail to down-regulate cell surface expression of HLA-I molecules.

## Methods

### Cells and recombinants

HaCaT, Hela and HEK-293T cells were grown in DMEM (Gibco) supplemented with 10% heat-inactivated fetal calf serum (FCS) and 1% penicillin/streptomycin. The two subclones of a human T cell leukaemia cell line CEM-C7 [29] and the calnexin-deficient CEM-NKR [30,31] were grown in RPMI 1640 (Gibco) with 10% heat-inactivated FCS and supplements. The coding region of HPV16 E5, an E5 alpha type protein [32], containing a HA-tag at the 5-end terminus and was cloned into the pCI vector (Promega) devoid of the starting methionine. Further, an AU1-tagged version of the E5 gene with codon usage adapted to the human relative synonymous codon usage preferences (Accession Number EF463082) was cloned into the pCDNA 3.1(+) vector (Invitrogen). A GFP-E5 fusion recombinant was synthesized by ligating the E5 wild-type coding region to the C-terminal end of the green fluorescence protein gene of the pEGFP vector [33].

Mutant recombinants were prepared by changing amino acids (QuickChange^® ^Site-Directed Mutagenesis Kit of Stratagene) in order to disrupt the putative transmembrane helix of each of the three domains of the E5 protein [34-36] without altering the length of the protein. All PCR-generated recombinants were confirmed by sequencing. Putative transmembrane domains of the E5 protein and the mutants were analysed using the TMHMM server version 2.0 [37,38].

### Transfections and confocal microscopy

Cells were transfected with Lipofectamine (HaCaT cells) or using the calcium phosphate method (Hela, HEK-293T). CEM-C7 and CEM-NKR cell lines were electroporated using 1×107 cells in 200 μl PBS, 10 μg DNA and setting the pulser to 220 Volt and 960 μFarad (Bio-Rad Gene-Pulser). Transfected CEM-C7 and CEM-NKR clones were selected with 0.8 mg/ml G418. For microscopy, transfected HaCaT cells were grown for 24 hours after transfection and then fixed with 4 % paraformaldehyde. Permeabilized, fixed cells were incubated with anti-AU1 (1:1000, Covance) or anti-calnexin (1:100, Santa Cruz), thoroughly washed and incubated with a secondary antibody labelled either with AlexaFluor^® ^488 or AlexaFluor^® ^594 (Molecular Probes). A LEICA laser scanning microscope (LEICA TCS SP) was used in all experiments.

### Immunoprecipitation

CEM-NKR and CEM-C7 transfectants were lysed with a modified RIPA buffer (150 mM NaCl, 1% NP-40, 0,5% sodium deoxycholate, 0,1% SDS, 1 mM EDTA, 1 mM EGTA, 50 mM Tris-HCl pH 8.0) supplemented with protease inhibitors. HEK-293T and Hela cells were transfected with the corresponding recombinants or with the empty vector. At 20–24 hours post transfection, the cells were lysed with a CHAPS buffer (0.2 M NaCl, 50 mM HEPES pH 7.5, 2% CHAPS) containing phosphatase- and proteinase-inhibitors for 20 min at 4°C. From the cell extracts 0.5 up to 1.5 mg proteins were immunoprecipitated with 2 μg of anti-AU1, anti-HA, anti-GFP or anti-calnexin. Immunoprecipitates were collected with protein G-sepharose, separated on acrylamide gels, blotted onto PVDF membranes and incubated with the appropriate antibodies. Reacting bands were revealed with the Western Lightning™ Chemiluminescence Reagent Plus (Perkin Elmer).

### Peptide translocation-assay

This assay was performed essentially as described [39] using the glycosylable peptide TNKTRIDGQY labeled with 125I by chloramine-T-catalyzed iodination. Cells were permeabilized with Streptolysin-O (Murex Diagnostics, Dartford, UK). 2 × 106 CEM-C7 or CEM-NKR cells were incubated with peptide and 10 mM ATP in 0.1 ml translocation buffer (130 mM KCl, 10 mM NaCl, 1 mM CaCl2, 2 mM EGTA 2 mM MgCl2, 5 mM HEPES pH 7.3) for 20 min at 37°C. Following lysis in 1% NP-40 (Sigma-Aldrich, Taufkirchen, Germany) the glycosylated peptide fraction was isolated with 30 μl concanavalin A-Sepharose slurry (Amersham-Pharmacia, Freiburg, Germany) and quantified by γ-counting. For control 5.0 mM EDTA was added instead of ATP.

### Flow cytometry and antibodies

HEK-293T cells were trypsinised 20 h post-transfection and incubated for 1 h in 37°C CO2-incubator to recover molecules expressed on the surface. CEM-NKR and CEM-C7 transfectants were stained with the HLA-A, B, C-reactive mAbs B9.12 [40]. Secondary antibodies were FITC-conjugated goat anti-mouse IgG (Dianova, 1:100) or PE-conjugated donkey anti-mouse IgG (Jackson ImmunoResearch Laboratories, 1:200). Incubations were performed in Eppendorf tubes for 45 min on ice in the dark, followed by two washes with ice-cold PBS/BSA. Cells were resuspended in 300 μl PBS/BSA and filtered in round-bottom polystyrene tubes (Greiner bio-one). Flow cytometry was performed with a FACSsort (Becton Dickinson).

### Statistical analysis

Analysis of FACS data and Kolmogorov-Smirnov statistics were performed with CellQuest™ software (BD Bioscience). Paired data were analysed with both the Wilcoxon Matched-Pairs Signed-Ranks Test -more conservative- and with the paired Student's t-test -less conservative. Inter-group comparisons were performed with both a Kruskal-Wallis test -more conservative- and with a one-way Analysis Of Variance (ANOVA) -less conservative. Differences below *p *value of 0.05 were considered significant.

## Results

### HPV16 E5 decreases surface expression of HLA-I molecules

**Figure 1:**
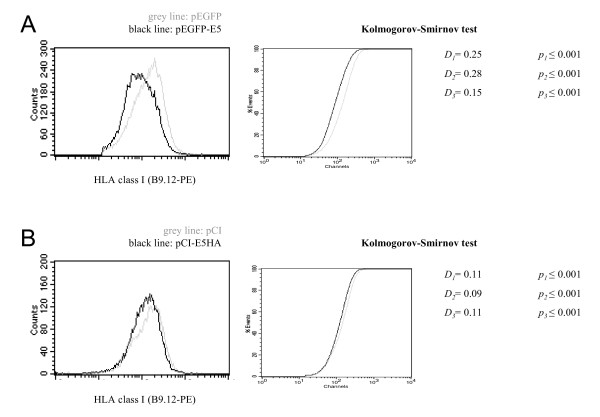
HPV16 E5 expression down-regulates HLA-I surface molecules. HEK-293T cells were transfected either with (*A*) pEGFP-HPV16-E5 or empty pEGFP vector, (*B*) pCI-HPV16-E5-HA or empty pCI vector. HLA-I molecules were then detected by immunostaining and flow cytometry using mouse monoclonal anti-HLA-A, B, C (mAb B9.12). Differences between the HLA-I surface expression levels were assessed by Kolmogorov-Smirnov test. This statistic defines the maximum vertical deviation between the two curves (pEGFP-E5 and GFP, pCI-E5-HA and pCI) as the statistic *D*. The *p *value of each single experiment was in all cases ≤ 0.001.

Experimental results have shown that BPV E5 as well as HPV16 E5 and HPV2 E5 proteins down-regulate surface expression of HLA-I molecules [22,24,41,42]. To evaluate this effect under our experimental conditions, we transfected pEGFP-HPV16-E5 or pCI-HPV16-E5-HA into HEK-293T cells and analysed cell surface expression of HLA-I by flow cytometry. Both constructs lead to a significant down-regulation of HLA-I surface expression (*p *≤ 0.001, Kolmogorov-Smirnov test, Fig. 1). For the pEGFP-HPV16-E5 and pEGFP constructs, the intracellular GFP-dependent fluorescence allowed us to gate GFP-expressing transfected cells making it possible to compare GFP-E5 with GFP positive populations in respect to their HLA-I signals (Fig. 1A). Further, in our hands the anti-HA antibody did not render sharp results differentiating transfected from untransfected cells. For this reason, the effects for the pCI-HPV16-E5-HA and pCI constructs were assessed by comparing total living cell populations (Fig. 1B). Since transfection efficiency never reached 100 %, reduction in relative values of the HLA-I surface expression tended to be more discrete in HPV16E5-HA than in pEGFP-HPV16-E5 transfected cells, leading to clearly significant though smaller values in the statistical analyses (Fig. 1A and 1B). These results therefore demonstrate that HPV16 E5 can down-regulate cell surface expression under our experimental conditions. Further, they also show that neither the small HA (10 amino acids) nor the large EGFP (239 amino acids) used for tagging the viral protein impairs the ability of HPV16 E5 to down-regulate HLA-I cell surface expression.

### HPV16 E5 expression reduces cell surface expression of HLA-I molecules in calnexin-expressing but not in calnexin-deficient cells

**Figure 2:**
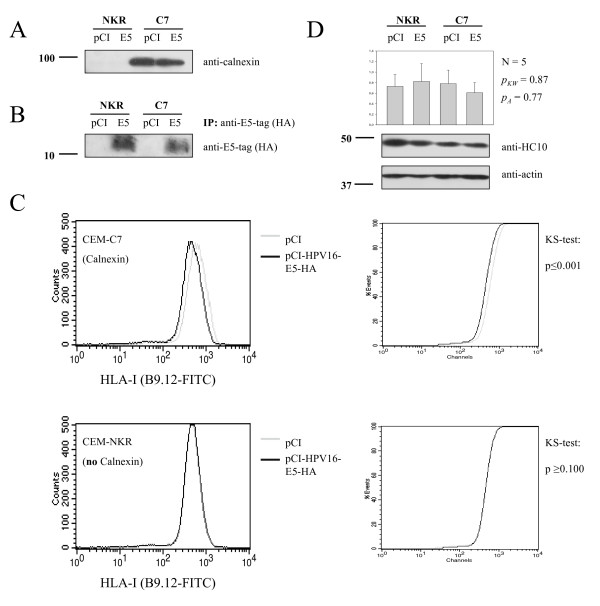
HPV16 E5 decreases HLA-I surface expression in calnexin-containing but not in calnexin-deficient cells. CEM-C7 (calnexin) and CEM-NKR (no calnexin) cells were stably transfected with pCI-HPV16-E5-HA or pCI empty vector. *A) *Calnexin is only expressed in CEM-C7 cells but not in CEM-NKR cells. *B) *E5-HA expression was analysed in each stable polyclone by immunoprecipitation and -blot using mouse monoclonal anti-HA Ab and 500 μg RIPA cell lysate. *C) *FACS analysis of CEM-NKR and CEM-C7 cells transfected with either the empty vector pCI or with pCI-E5-HA were stained with anti-HLA-A, B, C mAbs B9.12. E5 expression results in diminished HLA-I surface staining in cells expressing calnexin, but not in calnexin deficient cells. *D) *The upper part of the blot shown in A was incubated with anti-HC-10 antibodies (anti HLA-B, C). Incubation with anti-actin antibodies was performed as loading control. Columns represent average values (N = 5) and the error bars comprise the corresponding standard deviations. There were no differences between the total amounts of cellular HLA (N = 5; *pKW *= 0.87, Kruskal-Wallis test, and *pA *= 0.77, ANOVA). Molecular-mass markers (in kDa) are indicated in the left of the blots.

Since calnexin plays an important role in maturation of the HLA-I complex, we decided to analyze whether E5 affects HLA-I surface expression by a mechanism involving calnexin. We transfected CEM-NKR and CEM-C7 cells with pCI-HPV16-E5HA or empty pCI vector and selected clones stably expressing E5. CEM-NKR [31] is a variant of the leukaemia cell line CEM [43] known to be deficient in calnexin expression (Fig. 2A) [30]. First, we checked whether the permanent transfectants expressed E5 at similar amounts. Pooled clones of both CEM-NKR and CEM-C7 cells were analysed by immunoblotting for E5 expression. As shown in Fig. 2B no major difference in the expression level was found between both cells types. We then analysed surface expression of HLA-I molecules by flow cytometry, using two different anti-HLA-I antibodies. Whereas calnexin-expressing CEM-C7 transfected with the E5 protein contained clearly reduced amounts of surface HLA-I molecules (Fig. 2C, left panels, KS-test p ≤ 0.001), the calnexin-defficient CEM-NKR transfectants showed no differences in HLA-I surface expression between E5-expressing cells and controls (Fig. 2C, right panels, KS-test p ≥ 0.100).

To test whether this effect simply reflected the presence of different total amounts of HLA-I proteins in the cells, we analysed the total amount of HLA-I molecules in CEM-NKR and CEM-C7 cells by immunoblotting. As shown in Fig. 2D, no major differences in the HLA-I content between CEM-NKR and CEM-C7 cells were found when using total cellular protein extracts from both cell lines (N = 5, pKW = 0.87, Kruskal-Wallis test, pA = 0.77, ANOVA). The E5-mediated reduction in the HLA-I amount at the cell surface was thus not mediated by a lower total cellular content of HLA-I proteins in the CEM-C7 transfectants. These results therefore strongly suggest that E5 affects surface HLA-I expression by a mechanism that involves calnexin.

### HPV16 E5 does not influence the transport activity of TAP

**Figure 3:**
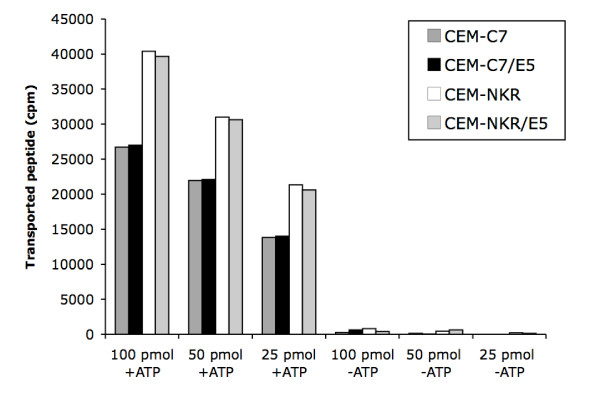
Transporter activity of TAP is not influenced by HPV16 E5. Streptolysin Opermeabilized calnexin-proficient CEM-C7 and calnexin-deficient CEM-NKR cells (38) were analysed in a peptide translocation/glyosylation assay using the indicated input quantities of the radioiodinated reporter peptide TNKTRIDGQY (glycosylation consensus site underlined) in the presence or absence of ATP. The glycosylated fraction, indicative of TAP-mediated ER transport, is isolated by concanavalin A Sepharose and quantitated by γ-counting. No significant differences could be detected between HPV16 E5-expressing cells and the control cells irrespective from the presence (CEM-C7) or absence (CEM-NKR) of calnexin.

Experimental evidence has been published showing that certain viruses target the TAP peptide transport as an effective strategy to reduce the availability of HLA-I-peptide complexes at the cell surface, thereby reducing the cellular susceptibility to CTL control and eventual lysis [44,45]. To determine whether HPV16 E5 interferes with the peptide transport activity of TAP in CEM cells, leading to the observed decrease in HLA-I surface expression, we applied a peptide translocation/glycosylation assay previously described [39]. As shown in Fig. 3, no differences in transport rates between E5 expressing and control cells were found, demonstrating that the transporter activity of TAP is not affected by HPV16 E5 expression in CEM-C7 and CEM-CEM-NKR transfectants.

### HPV16 E5 and calnexin can be co-immunoprecipitated from cellular extracts

**Figure 4:**
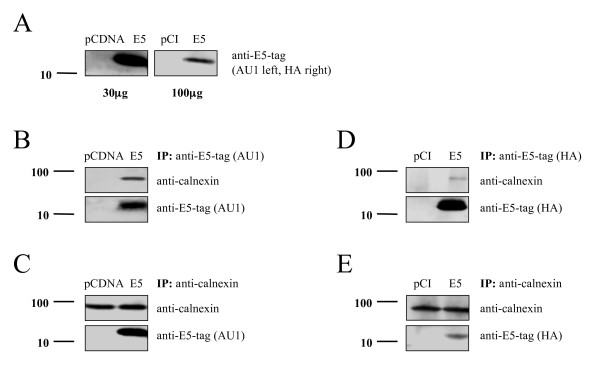
Calnexin interacts with the HPV16 E5 protein in cellular extracts. HEK-293T cells were transfected with AU1-tagged codon-optimised HPV16 E5, pCI-HPV16-E5-HA or corresponding empty vectors and lysed at 24 h posttransfection with CHAPS lysis buffer. *A) *Immunoblot showing the expression levels of the codon-optimised E5 gene (left panel) and of the viral E5 gene (right panel). Note the differences in the immunoreactivity signals despite the higher amount of total protein loaded in the non-optimised gene (100 μg vs 30 μg). *B) *Immunoprecipitations were performed using monoclonal anti-E5-tag (AU1) antibodies and proteins in the immune complexes were probed using anti-AU1 and anti-calnexin antibodies. *C) *Immunoprecipitations were performed using monoclonal anti-calnexin antibodies and proteins in the immune complexes were probed using anti-calnexin and anti-E5-tag (AU1) anti-bodies. *D) *Immunoprecipitations were performed using monoclonal anti-E5-tag (HA) antibodies and proteins in the immune complexes were probed using anti-HA and anti-calnexin antibodies. *E) *2 Immunoprecipitations were performed using monoclonal anti-calnexin antibodies and proteins in the immune complexes were probed using anti-calnexin and anti-E5-tag (HA) antibodies. Molecular-mass markers in kDa are indicated at the left of the blots.

To examine whether there is a physical interaction between E5 and calnexin, we transfected cells with HPV16 E5 and analysed whether calnexin and E5 could be co-immunoprecipitated. Since protein expression of the viral E5 gene is very weak in transfected cells, we prepared a codon-adapted version of the E5 sequence fitting to the codon usage preferences in humans, a procedure known to allow for increased protein expression of the protein in eukaryotic cells [46-48]. HEK-293T cells were transfected with the codon-adapted E5-coding DNA and protein expression levels were tested by Western blot. As shown in Fig. 4A (left) the codon-optimised E5 gene is well expressed in HEK-293T cells, some orders of magnitude above the expression achieved for the wild-type E5 gene (Fig. 4A, right). Cellular proteins were immunoprecipitated with antibodies against the AU1-tagged E5 protein, separated on SDS-PAGE, blotted, and the membrane was subsequently incubated with antibodies against calnexin. A band of 90 kDa apparent molecular mass corresponding to calnexin was identified in the immunoprecipitates, demonstrating that HPV16 E5 and calnexin could be co-immunoprecipitated in extracts of transfected cells (Fig. 4B). To further substantiate these results we performed the reverse experiment immunoprecipitating the extracts from transfected cells first with calnexin antibodies and then incubating the separated immunoprecipitates on the membrane with anti-E5-tag antibodies (anti-AU1). As shown in Fig. 4C, a reacting band of about 10 kDa was observed. This is the molecular mass found for HPV16 E5 when total cellular protein extracts were used for the immunoblots. These results demonstrate that HPV16 E5 and calnexin either directly interact *in vitro*. This interaction could also be reproduced when non-optimised viral E5-coding DNA (pCI-HPV16-E5-HA) was used for transfection (Fig. 4D and 4E), indicating that the effects did not arise from the higher amount of protein expressed from the codon-adapted version (Fig. 4A).

**Figure 5:**
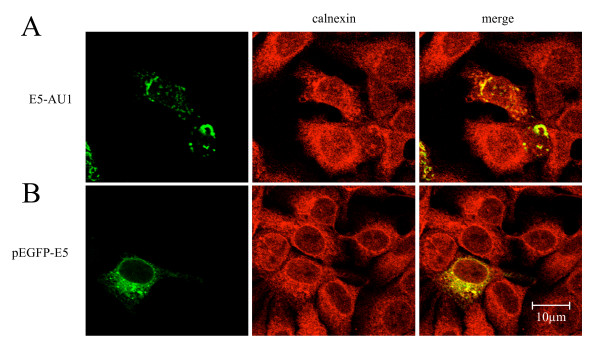
Co-localization of HPV16 E5 with calnexin. HaCaT cells were transfected with AU1- tagged codon-optimised E5 or pEGFP-E5 and analysed after 24 h by confocal laser scanning microscopy using a monoclonal anti-AU1 and/or polyclonal anti-calnexin Abs.

To further corroborate this finding at the intracellular level we next sought to demonstrate co-localization of both proteins in human keratinocytes expressing the E5 protein. HaCaT cells were transiently transfected with AU1-tagged codon-adapted E5 and co-localization with calnexin was analysed by laser confocal double immunofluorescence microscopy. As shown in Fig. 5A we observed a sharp colocalization of both proteins, confirming already published results for retroviral transduced keratinocytes [48]. Similar results were obtained when the GFP fusion protein was expressed instead of the AU1-tagged codon-optimised E5 protein (Fig. 5B), indicating that the subcellular localization of the E5 protein does not depend on the nature of the tag used to label E5.

### An intact hydrophobic region of HPV16 E5 is necessary for binding to calnexin

**Figure 6:**
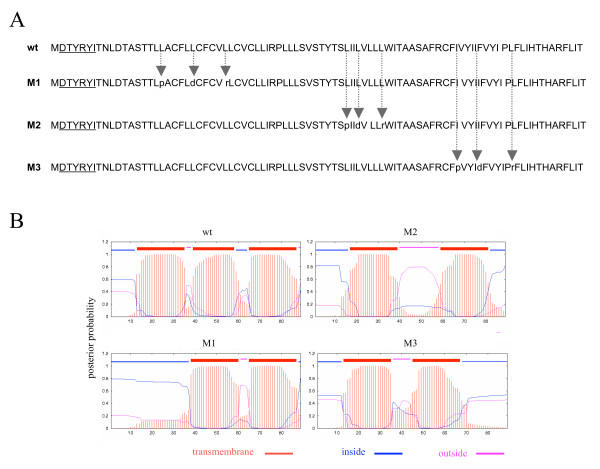
Transmembrane Hidden Markov Model posterior probabilities for the sequences of E5 and the mutants M1, M2 and M3. *A) *Amino acid sequence of the wild-type E5 protein and corresponding mutants. Aminoacids of the AU1-tag are underlined. Arrows show the position of exchanged amino acids. *B) *Analysis of the wild-type E5 and mutants using the TMHMM 2.0 algorithm (36, 37), showing the three hydrophobic regions predicted to be transmembrane domains, and the corresponding disruptions in the three mutants.

To analyze the characteristics of the E5-calnexin binding in more detail, we prepared a series of point mutants -M1, M2 and M3- in which we modified the E5 protein sequence, altering the hydrophobic profile and the local propensity to form helical structures. Leucine and/or isoleucine residues were mutated to proline, aspartate or arginines and then the resulting hydrophobic profile, propensity to helical structure and potential for stably spanning the cellular membrane were analysed and compared with those of the wild-type E5 protein (Fig. 6A, 6B). The point mutations were chosen so that they resulted respetively in the disruption of each of the three putative transmembrane helix within each of the three hydrophobic domains of the E5 protein, without changing the total protein length. All three mutants were based on the codon-optimised version of E5.

**Figure 7:**
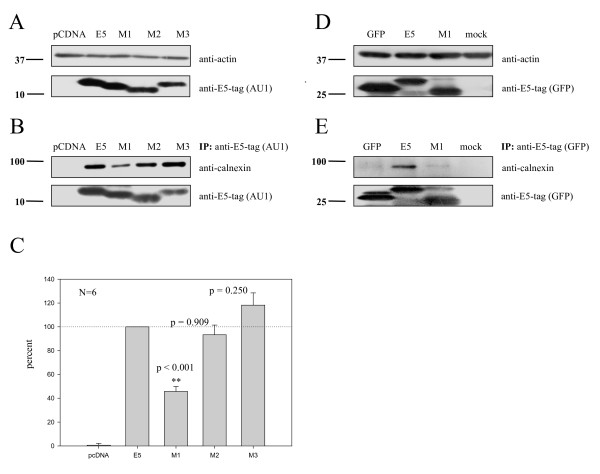
Mutant M1 binds less calnexin than wild-type E5 protein. HEK-293T cells were transfected with either (*A-C*) AU1-tagged codon-optimised HPV16 E5, the mutants M1, M2 and M3 or pcDNA 3.1 empty vector as control, (*D *and *E*) pEGFP-tagged HPV16 E5, mutant pEGFPM1, mock-control or pEGFP empty vector and lysed at 24 h posttransfection with CHAPS lysis buffer. *A) *Similar expression levels of all HPV16 E5 and the mutants M1, M2 and M3. *B) *Immunoprecipitations were performed using monoclonal anti-AU1, and proteins in the immune complex were detected using anti-AU1 and anti-calnexin. *C) *Quantification of co-precipitated calnexin for wild-type HPV16E5 protein, the mutants M1, M2, M3 and the vector control. The wild-type expression level was set to 100%. Data shown represent six independent experiments 2 plus standard errors of the mean. *P *values were calculated with paired two-tailed Student's *t*-test. *D) *Similar expression levels of pEGFP-HPV16-E5 and the mutant pEGFP-M1. *E) *Immunoprecipitations were performed using monoclonal anti-GFP, and proteins in the immune complex were detected using anti-GFP and anti-calnexin. Molecular-mass markers in kDa are indicated at the left of the blots.

To test whether the mutants M1, M2 and M3 were expressed at similar levels, HEK-293T cells were transfected with the original codon-optimised E5 sequences or with each of the mutants, and the protein content was analysed by immunoblotting. As shown in Fig. 7A, all recombinants showed similar levels of expression, being differences in SDS-PAGE migration attributable to the different hydrophobicity of the proteins.

To analyze the differential involvement of the each of the three E5 transmembrane domains in the interaction between E5 and calnexin, we performed immunoprecipitation experiments with the three mutants M1, M2 and M3 as described above. Protein extracts from transfected cells were immunoprecipitated with antibodies against the AU1 epitope, and the precipitates were analysed for calnexin content by immunoblotting. As shown in Fig. 7B, the original codon-optimized E5 protein and the mutants M2 and M3 co-precipitated calnexin to similar extents, whereas mutant M1 precipitated clearly reduced amounts of calnexin. To discard artefacts due to different inputs of antibody, protein G-sepharose or protein, the experiments were repeated six times. As shown in Fig. 7C mutant M1 co-precipitated calnexin to only 50 % of the levels precipitated by the wild-type and mutants M2 and M3. These results could be reproduced when non-optimised viral E5-coding DNA (pEGFP-HPV16-E5 and pEGFP-M1) was used for transfection instead of the codon-adapted E5-coding DNA (Fig. 7D and 7E). Taken together, these results strongly suggest that the first hydrophobic region of E5, i.e. the first putative transmembrane domain of the protein, is involved in the interaction with calnexin.

### Co-localization of HPV16 E5 and calnexin is dependent on the presence of the first hydrophobic domain of E5

The experiments described above indicate that the interacion between E5 and calnexin relies on the presence of an intact first hydrophobic region, and that this binding may be responsible for down-regulation of HLA-I expression. Should this be true, a reduction in co-localization between calnexin and mutant M1 would be expected in immunofluorescence experiments. In order to address this point, HaCaT cells were transfected with the three mutants M1, M2, and M3 and double immunofluorescence with anti-calnexin and anti tag antibodies was performed.

**Figure 8:**
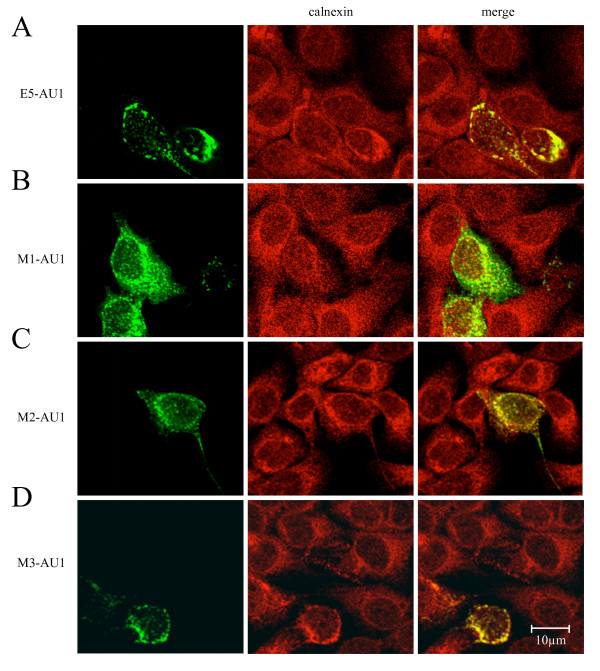
HPV16 E5, M2 and M3 mutants but not M1 mutant strongly co-localize with calnexin. HaCaT cells were transfected with *A) *AU1-tagged codon-optimised E5 or AU1- tagged codonoptimised E5 mutants M1 *B)*, M2 *C)*, and M3 *D) *and analysed after 24 h by confocal laser scanning microscopy using a monoclonal anti-AU1 and polyclonal anticalnexin antibodies.

As shown in Fig. 8, calnexin colocalized with the E5 protein expressed from the codonoptimized gene (Fig. 8A), as well as with the M2 and M3 mutants (Fig. 8C and 8D). In contrast, the disruption of the first helix in mutant M1 results in a change in the subcellular localisation of the protein, yielding a disperse and punctuate subcellular distribution, where only a partial co-localization with calnexin (Fig. 8B). These results are consistent with those found in the immunoprecipitation experiments and further confirm that the interaction of HPV16 E5 and calnexin requires a native, non-modified first transmembrane domain of the viral protein.

### Calnexin, HPV16 E5 and HLA form a trimeric complex

**Figure 9:**
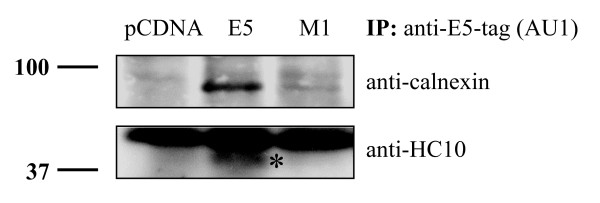
HPV16 E5 forms a ternary complex with calnexin and the HLA-I heavy chain. HeLa cells were transiently transfected with AU1-tagged codon-optimised HPV16 E5, M1, or empty vector. 24 h later CHAPS lysates were immunoprecipitated with antibodies against the E5-tag (anti-AU1). Precipitated immune complexes were separated by SDS-PAGE and Western blotted using anti-calnexin and anti-HLA-B, -C mAb (HC10), respectively (band marked with *). Molecular-mass markers in kDa are indicated at the left of the blots.

Recent results have shown that HPV16 E5 may co-precipitate with the heavy chain of HLA-I [21]. In the light of our results presented above, and together with the fact that HLA-I and calnexin associate during HLA maturation, we hypothesized that the formation of a trimeric complex between HLA-I heavy chain, calnexin and E5 might be involved in the retention of HLA-I in the ER/Golgi apparatus of the cells expressing E5. To address this question, HeLa cells were transfected with AU1-tagged codon-optimised E5 or with mutant M1, and protein extracts were immunoprecipitated with anti-AU1. Immunoprecipitates separated in SDS-PAGE, were blotted onto PVDF membrane and probed either with anti-HC10, recognizing HLA-B, C heavy chains [49], or with anti-calnexin antibodies. As shown in Fig. 9, both HLA-I heavy chain and calnexin could be co-immunoprecipitated with anti-AU1 antibodies, which target E5. More important, the E5 mutant M1 previously shown to be deficient in immunoprecipitation of calnexin, also failed to co-precipitate the HLA-I heavy chain. These results demonstrate that HPV16 E5 forms a complex with calnexin and HLA-I heavy chain and that this complex depends on the interaction of the first hydrophobic region of E5 with calnexin.

### Mutant M1 is not able to down-regulate HLA-I cell surface expression in the same extent that wild type HPV16 E5 does

**Figure 10:**
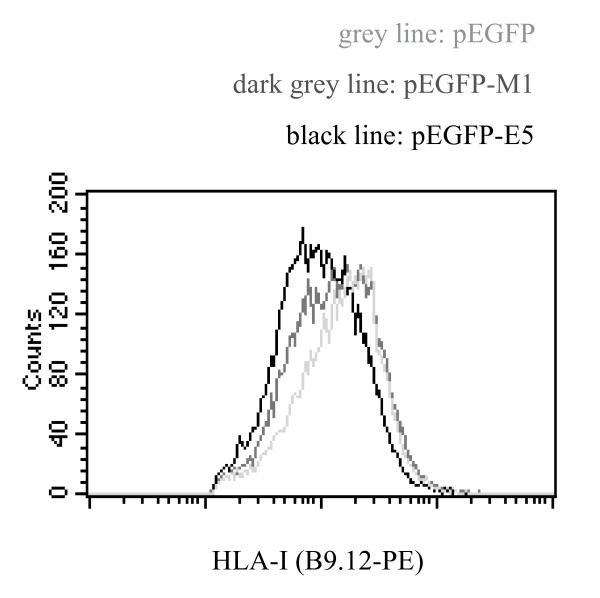
E5 mutant M1 down-regulates surface expression of HLA-I to a lesser extent than the E5 protein does. HEK-293T cells were transfected either with pEGFP-HPV16- E5, -M1, or empty pEGFP vector. HLA-I molecules were detected by immunostaining and flow cytometry using mouse monoclonal anti-B9.12 Ab. Results for one representative experiment out of six are shown. Statistic analysis is shown in Table 1.

Since the experiments shown above demonstrate that mutation of the first putative transmembrane helix of E5 results in the loss of binding to calnexin, we addressed the question whether this loss correlates with the failure to down-regulate HLA-I surface expression. HEK-293T cells were transfected with the wild-type pEGFP-E5, mutant pEGFP-M1 or pEGFP empty vector and the amount of HLA-I expression at the cell surface was determined by FACS analysis. While wild-type E5 expression resulted in HLA-I down-regulation at the plasma membrane (Figs. 1 and 2C, Fig. 10), this effect was not observed when the cells expressed the E5 mutant M1 (Fig. 10). To substantiate this result, we did the experiment six times and analysed the median values of HLA-I surface expression in the transfected cells (for statistical analysis, see Table 1). Whereas the wild type E5 protein was able to down-regulate HLA-I surface expression down to 65% (median of six experiments), the median HLA-I staining of HEK-293T transfected with the E5 mutant M1 was 82% (median of six experiments) as compared with HEK-293T control transfectants (N = 6, pW = 0.0313, Wilcoxon matched-pairs signed ranks test, pST = 0.009, paired Student's t-test).

Taken together, our results strongly indicate i) that E5-mediated down-regulation of HLA-I surface expression proceeds through the formation of a ternary complex between E5, calnexin and the heavy chain of HLA-I; ii) that the disruption of the first transmembrane domain of HPV16 E5 modifies the subcellular distribution of the protein; and iii) that the disruption of the first transmembrane domain of HPV16 E5 prevents the interaction, colocalisation and immunoprecipitation of the viral protein with calnexin, and also of that with the heavy chain of HLA-I.

## Discussion

Eukaryotic cells respond to viral infection by activating mechanisms aiming to abortion of the infection through hindering of viral protein expression, virus maturation or virus release, while viruses have developed during evolution molecular countermeasures to escape from these cellular controls. One of these viral strategies leads to a reduction in the adaptive immunoresponses of the host by reducing the exposure of the infected cells to immune surveillance. Reduced surface expression of HLA-I has been described upon expression of HPV16 E5 or HPV2 E5 proteins [22,42], but the molecular mechanisms responsible for the decrease of HLA-I on the cell surface have not yet been elucidated. In this report we present experimental evidence demonstrating that HPV16 E5 down-regulates HLA-I surface expression by a calnexin-mediated mechanism. Using transient and stably transfected cells, we have shown that HPV16 E5 is able to reduce HLA-I surface expression in calnexin-containing cells, but not in a calnexin-deficient cell line. Published reports have described that the heavy chain of HLA-I molecules and HPV16 E5 could be co-precipitated [21], suggesting that this binding might be involved in HLA-I down-regulation. Nevertheless, our results point to the binding of E5 to calnexin as the critical molecular event directly involved in HLA down-regulation. Expression of E5 in CEM-C7 cells, which constitutively express calnexin, results in a decreased amount of HLA-I at the cell surface, but no down-regulation was observed in CEM-NKR cells devoid of calnexin (see Fig. 2C). Since both cell types CEM-C7 and CEM-NKR contain similar amounts of HLA-I molecules (Fig. 2D and see [30]) it seems unlikely that a putative binding of HPV16 E5 to the HLA-I heavy chain alone could be solely responsible for the decreased surface expression of HLA-I proteins in CEM-C7 cells.

Regarding other viruses, such as herpes simplex virus and cytomegalovirus, it has been shown that they target the transporter associated with antigen processing (TAP) in order to down-regulate HLA-I surface expression [50,51]. In PVes it has been demonstrated that purified HPV11 E7 protein is able to inhibit ATP-dependent peptide transport into the lumen of the ER in vitro [52]. In this context, our peptide translocation-assay results show that HPV16 E5 does not influence the transport of antigen peptides from the cytosol to the ER. Thus, the data here presented suggest that HPV16 E5 does not target the TAP transporter activity to control surface expression of HLA-I molecules.

Our co-immunoprecipitation experiments using either antibodies against different tagged versions of the E5 protein or against calnexin demonstrate that HPV16 E5 associates with calnexin *in vitro*. The biological significance of this interaction is further supported by the previously described intracellular co-localization of calnexin and HPV16 E5 [48], that we confirmed in this report.

Upon interaction between the first and the third hydrophobic segments [53], HPV16 E5 could be organized as a transmembrane protein with three putative transmembrane helices [54]. In the present work we have introduced specific point mutations in this E5 gene, selectively targeting local hydrophobicity and propensity towards helix conformation in each of the three predicted transmembrane helices of the HPV16 E5 protein [32]. These point mutations result in the selective and individual disruption of each helix without altering the overall length of the protein. Our results reveal that the first hydrophobic helix is mainly responsible for HPV16 E5 subcellular localisation and concomitantly for colocalisation between HPV16 E5 and calnexin. Mutant M1 -with the first putative transmembrane helix being disrupted- was able to bind reduced amounts of calnexin in immunoprecipitation assays, while co-localizing only weakly with calnexin in transfected cells. In addition, M1 transfectants did not down-regulate surface expression of HLA-I in the same extent than wild-type E5. Together both results suggest that i) the first putative transmembrane domain of HPV16 E5 is responsible for the HPV16 E5 localisation; ii) the interaction of HPV16 E5 and calnexin depends on the integrity of the first putative transmembrane domain; iii) the effect of HPV16 E5 on HLA-I surface expression strongly depends on the integrity of the first putative transmembrane domain and on the subsequent interaction between HPV16 E5 and calnexin.

The definitive finding presented here is the existence of a ternary protein complex of HPV16 E5, calnexin, and the heavy chain of HLA-I molecules. The formation of this complex depends on the presence of the first predicted transmembrane domain of HPV16 E5. Since the dimer calnexin-HLA is a natural step in the antigen processing route, it can be hypothesized that HPV16 E5 binds to the calnexin-HLA-I complex and that this binding blocks further trafficking of the HLA-I complex to the plasma membrane, leading instead to its accumulation in the ER/Golgi of the infected cell. A direct binding of E5 to the heavy chain of HLA-I seems under the light of our results improbable. This is further supported by our findings using calnexin-deficient cells lines. Although both cell types, calnexin-containing and calnexin-deficient, express similar amounts of heavy chain HLA-I, the E5-mediated reduction of surface HLA-I becomes evident exclusively in calnexin-containing cells.

The interaction between E5 and calnexin could be demonstrated in cells transfected with the codon-adapted version of the gene, and also in cells transfected with the wild-type gene. This association is therefore independent from the effective amount of E5 protein expressed, and cannot be due to a very large overexpression from the optimised version of the gene. This is not a trivial result, as it has been shown that codon usage optimization can lead to changes in the phenotype associated with protein expression [55,56].

## Conclusion

In summary, our results support a model for the E5-mediated HLA-I surface downregulation in which the viral protein interacts with calnexin, finally leading to an E5- calnexin-HLA-I heavy chain ternary complex unable to be further transported to the cell surface.

## Authors' contributions

MG performed molecular biology, cell biology, confocal microscopy and flow citometry experiments, and drafted the manuscript. IGB participated in the design of the research concept and in mutant design, performed statistical analyses and drafted the manuscript. FM performed the peptide translocation assay. AA conceived and supervised the study and drafted the manuscript. PT collaborated in the supervision of the study and helped draft the manuscript. All authors have read and approved the final manuscript.

**Table 1:** E5 mutant M1 down-regulates surface expression of HLA-I to a lesser extent than the E5 protein does.

	E5-GFP	***p*- value**^c^	M1-GFP		E5-GFP	***p*- value**^c^	M1-GFP
HLA-	57.77%	***t- *test**	69.78%	KS-*D*^b^	0.29	***t- *test**	0.20
surface	67.92%	**0.009**	75.67%		0.17	**0.005**	0.13
expression^a^	75.67%		94.75%		0.15		0.04
	69.16%	**Wilcoxon**	82.04%		0.20	**Wilcoxon**	0.11
	59.35%	**0.0313**	82.78%		0.25	**0.0313**	0.10
	62.08%		100.90%		0.23		0.02
Median	65.00%		82.41%	Median	0.215		0.105
Range	58%-76%		70%-100%	Range	0.17–0.29		0.02–0.2

^a^Values of pEGFP-E5 and pEGFP-M1 were normalized to the values of the pEGFP-control (E5-GFP, M1-GFP), which was set as 100% expression of HLA-I surface expression.

^b^Kolmogorov-Smirnov (KS) test was performed with CellQuest™ software (BD Bioscience). This statistic defines the maximum vertical deviation between the two curves (pEGFP-E5 and pEGFP-control or pEGFP-M1 and pEGFP-control) as the statistic *D*. The *p *value of each single experiment was ≤ 0.001.

^c^Paired two tailed paired student's *t*-test and Wilcoxon matched-pairs signed-ranks test values for the raw percentages of immunorreactive cells (left column) and for the *D *statistic (right column). The E5 mutant M1 does not affect HLA-I expression in the same extent than the original E5 protein does (*p *< 0.05 in all cases).
